# Supplementary material for: Reliability and validity of the Perceived Stress Scale in Bangladesh
Source: PLoS One. 2022 Oct 27;17(10):e0276837. doi: 10.1371/journal.pone.0276837 (PMC9612580; doi:10.1371/journal.pone.0276837)
Supplement: Attachment — Submitted filename: Response to Editors Comments.docx [file pone.0276837.s001.docx]

Dear Editor,

I have revised the document as per suggestion and have completed the journal requirements suggestion from you. Please find below the response to the additional comments.

Additional Editor Comments (if provided):


1. In general terms, it seems to me that the theoretical framework is adequately written, within what is to be expected for a psychometric article, although I believe that the arguments for the relevance of developing validity evidence for this population could be better developed. In addition, there is a lack of theoretical justification of the variables used for the evidence of validity in relation to other variables.
**Response:** Thank you for this comment. A new sentence added in introduction. Some details on the justification of the validation variables are included in the last paragraph of data analysis section.

2. In the methodological aspects, I suggest not using exploratory factor analysis, since there is a well-defined model and, in case it is performed, not using the principal components estimation method, since this estimation is not a factor estimation, since it corresponds to formative (not reflective) models. Another even more important weakness is to perform the exploratory and confirmatory analysis with the same sample, since it is redundant and any spurious structure would be confirmed by mere capitalization of chance (if this double process is done, different samples should be used), producing an overfitting. To avoid this, if sufficient sample size is not available, I suggest considering the application of ESEM, instead of EFA and CFA.

**Response:** Thank you for this suggestion. I have removed the EFA part of analysis. I strongly agree that this is redundant for this context.

3. All these aspects mentioned correspond to suggestions that, most probably, would be quickly noticed by the reviewers, but all of them can be corrected relatively easily. What concerns me, given that it requires extending and modifying the sampling, is the sample size, but, above all, the lack of representativeness of the sample, since it claims to refer to the general population, but includes almost a third of the LGBTQ population (with which a particular psychometric study has already been conducted), which is evidenced as a sample recycling that is not consistent with the aims of the study. Although in these disciplines it is very rare to have random samples, it is necessary to give some guarantees that the sample is reasonably close to the population in which the instrument is intended to be used, which is not met here, so it is necessary to conduct additional sampling in order to make the study feasible.

Therefore, I have decided not to send the study for review, unless they complement the sample.

**Response:** Please note that ‘No data from LGBT population’ is used in this analysis. All the data are from general population. Part of the data was collected as comparison group for LGBT as no comparable normative data from general population was available at that time. Moreover, none of these data were published in any other articles. Therefore, observation regarding sample recycling is inaccurate. Slight modification of text is made in the method section to reduce confusion.

I agree completely that this is not at all a complete representation of general population of the country. However, we try tried to get closer to that by collecting data from all the Eight divisions of Bangladesh. Achieving more that that would require huge resources reducing feasibility of the study. A sentence has been added to the conclusion section.

4. Additionally, for the review of psychometric studies, it is required that they make the data available, even if they are only those referring to the items of the instruments (which would not interfere with other subsequent or parallel studies), so that the reviewers can review the adjustments of the measurement models if required.

**Response:** Thank you for making it clearer, now I have made the data publicly available.
