# Supplementary material for: Reliability and validity of the Perceived Stress Scale in Bangladesh
Source: PLoS One. 2022 Oct 27;17(10):e0276837. doi: 10.1371/journal.pone.0276837 (PMC9612580; doi:10.1371/journal.pone.0276837)
Supplement: Attachment — Submitted filename: Responses to reviewer.docx [file pone.0276837.s002.docx]

**Responses to the Reviewer’s comments**

Thank you for reviewing this manuscript and making suggestion to improve the manuscript (PONE-D-21-21312R1; Reliability and validity of the Perceived Stress Scale in Bangladesh). Please find my responses after each of your suggestions (listed in numbers).

1. I think it would be important to explain better, why theoretically what the PSS-10 measures should be related to what the GHQ-28 measures, so that the reader has more clarity regarding the nomological network around the PSS-10.

**Response:** Thank you for this suggestion, I have added a sentence in the manuscript to address this, “*It may be noted here that the underlying focuses of the GHQ-28 are the appearance of distressing phenomenon and the inability to carry out normal functions, which logically are connected to the perception of stress measured by the PSS 10.”* (line 151-153, page 8)

2. Also, it would be advisable to include the operational definition of the factors represented by the PSS-10, in order to have a better understanding of the meaning of the results obtained.

**Response:** I agree that this would be useful however, I did not find any operational definition from the authors who first coined these two factors.

3. On the other hand, there are some assertions in the paper that could be misinterpreted. For example, in the instruments part it says "The SRQ-20 has been validated in several countries, including Bangladesh [25], and is widely used as a screening and research tool", this could lead to believe that "the tests are valid", however, as explained in more detail by the AERA, APA, & NCME (2014), the tests are not valid, but the use and interpretation of the scores could be or not.

**Response:** This was a new insight for me, thank you for your comment. The sentence has now been revised to avoid the misinterpretations, “*Validation studies on the SRQ-20 have been carried out in several countries, including Bangladesh”.* (line 103-104, page 6)

I have also revised another sentences based on this suggestion, “*validation studies on the PSS have also been carried out in different subpopulations*” (line 29-30, page 3)

4. A little more precision is needed regarding the wording of the results obtained from Cronbach's Alpha coefficient. I recommend reading Cho and Kim (2015) or Hoekstra et al., (2018), where they explain some common errors in the interpretation of this coefficient.

**Response:** This is very useful suggestion. Thank you for the recommended articles, they explained much more details on the misinterpretations of Cronbach's Alpha. I have rephrased some sentences to avoid such misinterpretations.

“The reported internal consistency of the scale indicated by Cronbach’s alpha ranged from.71 to .91” (line 83-84, page 5)

“Cronbach’s alpha was used as an indicator for internal consistency of the scale. For the Bengali PSS-10 full scale alpha was .715” (line 180-181, page 9)

The word “proved” has been replaced with “suggested”, “The findings suggested that the PSS-10 and its two factors are internally consistent” (line 231, page 12)

5. Finally, taking into consideration that Bangladesh is a country with more than 150 million inhabitants and that the presented study does not have randomized sampling, I recommend being more careful about generalizing the results.

**Response:** Although a caution was reflected at the end of discussion (“*Although the lack of randomization and the absence of data on the response rate raise concerns regarding the generalizability of the findings, the inclusion of countrywide participants with different socio-demographic characteristics makes the results useful.”;* line 251-254, page 12*)* a new sentence has now been added in the conclusion section, “*However, the result can never be generalized and caution should be taken before utilizing and interpreting scale scores from the general population based on current findings*.” (line 264-266, page 13)

Additionally, in the abstract the word “established” has been replaced with “suggest” in sentence “*this study suggest the PSS-10 as a valid and reliable instrument*“ (line 11-12, page 2)
