# Supplementary material for: Reliability and validity of the Perceived Stress Scale in Bangladesh
Source: PLoS One. 2022 Oct 27;17(10):e0276837. doi: 10.1371/journal.pone.0276837 (PMC9612580; doi:10.1371/journal.pone.0276837)
Supplement: Attachment — Submitted filename: peer review.docx [file pone.0276837.s003.docx]

The purpose of the research was about the psychometric properties of the Bengali PSS-10 demonstrated in this study suggest the PSS-10 as a valid and reliable instrument for use in Bangladesh and among Bengali-speaking populations.

Strengths of the research several method used to test the reliability of the instruments with strong Cronbach’s alpha and high correlation score for test retest. A appropriate sample size for the study was obtained. Enough evidence was provided for conclusions. The literature review supported the topic and hypothesis. The language was clear and easy to follow. Tables and figures are clear and support findings. While the study appears to be sound, the author should justify his choice of method.

One of the weaknesses of the study is that abstract need to be clear in terms of the main research questions. The author should rewrite the abstract section to support the study purposes with each hypothesis ideas and major finding clearly. Limitation of the study not enough.

Reviewer: Reham Bakhsh
